# Vaginal Microbiota, Genital Inflammation and Extracellular Matrix Remodelling Collagenase: MMP-9 in Pregnant Women With HIV, a Potential Preterm Birth Mechanism Warranting Further Exploration

**DOI:** 10.3389/fcimb.2021.750103

**Published:** 2021-11-29

**Authors:** Charlotte-Eve S. Short, Rachael A. Quinlan, Xuan Wang, Veronica Georgiana Preda, Ann Smith, Julian R. Marchesi, Yooni S. Lee, David A. MacIntyre, Phillip R. Bennett, Graham P. Taylor

**Affiliations:** ^1^ Section of Virology, Department of Infectious Disease, Faculty of Medicine, Imperial College London, London, United Kingdom; ^2^ March of Dimes Prematurity Research Centre, Division of Development and Reproductive Biology, Department of Metabolism, Digestion and Reproduction, Faculty of Medicine, Imperial College London, London, United Kingdom; ^3^ Faculty of Health and Applied Sciences, University West of England, Bristol, United Kingdom; ^4^ Division of Digestive Diseases, Department of Metabolism, Digestion and Reproduction, Faculty of Medicine, Imperial College London, London, United Kingdom

**Keywords:** HIV - human immunodeficiency virus, preterm (birth), metalloproteinase, microbiome and dysbiosis, *Gardnerella* species

## Abstract

**Background:**

Pregnant women living with HIV infection (PWLWH) have elevated rates of preterm birth (PTB) in which HIV and cART are implicated. PWLWH also have a high prevalence of adverse vaginal microbiota, which associate with genital tract inflammation. The mechanism underlying PTB in PWLWH is unknown. We present the first data in PWLWH on genital-tract matrix-metalloproteinase-9(MMP-9), an important collagenase implicated in labour onset, and tissue inhibitor of metalloproteinases-1(TIMP-1) and explore correlations with local inflammation and vaginal bacteria.

**Material and Methods:**

Cervical vaginal fluid (CVF) collected by a soft cup and high vaginal swabs (HVS) were obtained from PWLWH and HIV uninfected pregnant women (HUPW) at three antenatal time points. Maternal characteristics, combination antiretroviral therapy (cART) exposure, and pregnancy outcome were recorded. Concentrations of MMP-9, TIMP-1 and ten cytokines were measured by immunoassays. Vaginal microbiota composition was determined through 16S rRNA amplicon sequencing. MMP-9, TIMP-1 and cytokine concentrations were compared by HIV status, cART, and prematurity and in PWLWH correlations with polymorphonuclear leucocytes, cytokines and bacterial genera were explored.

**Results:**

CVF was available for 50 PWLWH (108 samples) and 12 HUPW (20 samples) between gestation weeks 14-38. Thirty-six PWLWH conceived on cART and 14 initiated post-conception. There were five and one PTB outcomes in PWLWH and HUPW respectively. PWLWH had higher mean CVF concentrations of MMP-9 (p<0.001) and TIMP-1 (p=0.035) in the second trimester compared with HUPW with a similar trend in the third trimester. PWLWH also had higher CVF values of cytokines: IL-1β, IL-8, IL-12 and TNF-α in both trimesters compared to HUPW (p ≤ 0.003). In PWLWH, MMP-9 positively correlated with TIMP-1 (r=0.31, p=0.002) and CVF polymorphonuclear leucocytes (r=0.57, p=0.02). Correlations were observed between MMP-9 and three cytokines: IL-1β (r=0.61), IL-8 (r=0.57) and TNF-α (r=0.64), p<0.001, similarly for TIMP-1. Abundance of anaerobic pathobionts correlated with MMP-9: *Gardnerella* (r=0.44, p<0.001), *Atopobium* (r=0.33, p=0.005), and *Prevotella* genera (r=0.39, p<0.001). Conversely proportion of *Lactobacillus* genera negatively correlated with MMP-9 (rho=-0.46, p<0.001). MMP-9/TIMP-1 ratio increased with gestational age at sampling in PWLWH, but this was no longer significant after adjusting for confounders and no difference by prematurity was observed in this sub-study.

**Conclusions:**

Here we show strong correlations of MMP-9 to genital tract inflammation and sub-optimal bacterial genera in PWLWH indicating the ascending genital tract infection pathway may be a contributory mechanism to the high risk of PTB.

## Introduction

Pregnant women living with HIV infection (PWLWH) are disproportionately affected by PTB outcomes, with some cohorts experiencing two to four-fold the risk observed in the general population (Thorne et al., 2004; Short and Taylor, 2014; Wedi et al., 2016). The aetiology of this phenomenon is unclear but the successful use of combination antiretroviral therapy (cART) to prevent mother to child transmission of HIV (PMTCT) has not had the same impact on high rates of PTB and may increase risk of this complication (Thorne et al., 2004).

Literature around this obstetric complication in PWLWH suggest that it is the result of a complex interplay of high background risk factors for PTB e.g. African and Caribbean ethnicity (Tuomala et al., 2005; Li et al., 2019), anaemia (Chen et al., 2012), low body mass index (BMI) (Young et al., 2012), hypertension (Chen et al., 2012) and pre-eclampsia (Wimalasundera et al., 2002), cART exposure (class of third drug e.g. protease inhibitors and timing in relation to conception) (C Short and Taylor, 2014) and infection factors. The latter include advanced HIV disease (Slyker et al., 2014; Wedi et al., 2016; Favarato et al., 2018), intercurrent co-infections with bacterial vaginosis (Taha, 1999; Slyker et al., 2014), sexual transmitted infections (Slyker et al., 2014; Shava et al., 2019), other pathogens e.g. cytomegalovirus, malaria (McDonald et al., 2019; Moraka et al., 2019), and higher risk and severity of chorioamnionitis (Goldenberg et al., 2006; Ategeka et al., 2019; Obimbo et al., 2019).

Preterm birth in the general population is an umbrella syndrome with multiple aetiologies (Goldenberg et al., 2008). Ascending genital tract infection is thought to be a significant factor in premature rupture of membranes (Brown et al., 2018) and spontaneous preterm labour (Elovitz et al., 2019) and potentially impair placentation, all effected through changes in maternal immune surveillance and tolerance (Mor et al., 2017). The pathologic mechanism of PTB in PWLWH is unclear. We hypothesize the elevated risk of PTB is the result of an inflammatory process driven by alternations in: the balance of cytokines at the maternal foetal interface (Fiore et al., 2006; Short and Taylor, 2014; Short et al., 2021) and dysregulation of immune activation of maternal T cells (Short et al., 2017) to promote local immune cell infiltration in the female reproductive tract; both of which are influenced by local vaginal microbiota composition (Short et al., 2013; Anahtar et al., 2015; Short et al., 2021).

We and others have described that PWLWH in European and African settings have a microbiota either dominated by *Lactobacillus iners* or with a diverse anaerobic bacteria community structure (Price et al., 2019; Gudza-Mugabe et al., 2020; Short et al., 2021). *Lactobacillus* single species dominance in pregnancy, particularly *L. crispatus*, is associated with term birth outcomes whereas *L. iners* species dominance, the least stable *Lactobacillus* community structure, and mixed anaerobes are associated with PTB (Kindinger et al., 2017; Brown et al., 2018; Elovitz et al., 2019; Fettweis et al., 2019). Within our London HIV PTB study cohort all PTB occurred in women with these adverse vaginal microbiota groups (Short et al., 2021). How these bacteria exert their effects on downstream pathways to trigger labour is not fully elucidated. Pilot data from our group exploring differentially expressed cervicovaginal immune proteins by HIV status using proteomic profiler arrays indicated that matrix metalloproteinase 9 (MMP-9), extracellular matrix metalloproteinase protein inducer (EMMPRIN) and neutrophil gelatinase B-associated lipocalin (NGAL) were significantly upregulated compared to HIV uninfected pregnant women (HUPW) (Short, 2019).

MMP-9 (also known as gelatinase B) is a zinc dependent endopeptidase involved in extracellular matrix (ECM) remodelling of type IV collagen (and to lesser extent type V) and elastin that is part of several physiological processes including uteroplacental re-modelling in reproduction, cell migration (particularly neutrophils), angiogenesis and wound healing(Juanjuan Chen and Khalil, 2017). It is known to be upregulated in membrane rupture, placental detachment and myometrial contractility in term and preterm labour (Weiss et al., 2007; Sundrani et al., 2012; Ulrich et al., 2019). MMP-9 expression occurs in multiple cell types, many of which are found at the maternal-foetal interface although the source of MMP-9 in the events leading to labour is not fully understood. Its expression is thought to be regulated by cytokines: IL-1β, IL-8, IL-10 and TNF-α, prostaglandins (Peltier, 2003; Padron et al., 2020), and tissue inhibitor metalloproteinase-1 (TIMP-1)(Juanjuan Chen and Khalil, 2017). Much of the available data on MMP-9 in pregnancy are derived from plasma and amniotic fluid samples, tissue explants, cell lines and rat models with minimal data on the cervical and lower genital compartments (Choi et al., 2009; Becher et al., 2010). Human data exist on the relationship between MMP-9 and intra-amniotic infection but the direct relationship with vaginal microbiota as the source are scant (Locksmith et al., 2001; Kindinger et al., 2016; Padron et al., 2020) and non-existent in PWLWH.

Here we explore MMP-9, TIMP-1 and cytokine concentrations, and local polymorphonuclear leucocytes in a subgroup of PWLWH from the London HIV PTB study for whom cervicovaginal fluid (CVF) and high vaginal bacterial metataxonomic data were available and examine the association with lower genital tract microbiota composition. The full characterization of the vaginal microbiota of PWLWH from the London HIV Preterm birth study has been described previously (Short et al., 2021). These data have been used for correlation analyses with CVF MMP-9, TIMP-1 and cytokine concentrations.

## Methods

### Study Design and Setting

The London HIV PTB study has been described previously (Short et al., 2021). PWLWH and HUPW were prospectively recruited between 2013-2017 at eleven tertiary care hospitals across London. The study was approved by the NHS Health Research Authority National Research Ethics Service (NRES) (Ref 13/LO/0107). The London HIV PTB study is an exploratory hypothesis generating pilot study and thus no sample size calculation has been performed. This is a sub-analysis of the main study of the group of women who provided lower female genital tract (FGT) samples.

#### Participants and Sample Collection

Exclusion criteria were: <18 years old, inability to give written informed consent, current injecting drug use, multiple gestation pregnancy and *in vitro* fertilization. All women not previously known to be living with HIV infection underwent routine antenatal screening with fourth generation combined HIV antibody and p24 antigen tests and syphilis serology in the first trimester. For PWLWH screening for gonorrhea and chlamydial infection was routinely offered as per national guidelines. Gestational age was determined by obstetric ultrasound at 10-14 weeks. Maternal demographics were recorded and for PWLWH: ART regimen, timing of cART exposure in relation to conception and immunovirological parameters were also documented. Gestational age at delivery, mode of delivery and any obstetric complications were recorded. Preterm birth was defined as delivery <37 completed weeks gestation.

Participants were invited to undergo lower FGT sampling at three time points across the second and third trimesters: 14.0-21.9 weeks; 22.0-27.9 weeks; 28.0-38.0 weeks. Clinician or self-taken high vaginal swab [HVS (BBL ™ CultureSwab™ MaxV Liquid amies swab)] and paired menstrual soft cup (Instead^®^, Evofem Ltd™) samples were obtained. Soft cups were retained for a minimum of five minutes prior to removal. All FGT samples were kept on ice and stored at -80°C within two hours of collection until further processing.

### Quantification of CVF MMP-9, TIMP-1, Cytokines and Polymorphonuclear Leucocytes

CVF was thawed and extracted from the soft cup as previously described (Short et al., 2018) and diluted in an extraction buffer containing a protease inhibitor cocktail (Castle et al., 2004) in a dynamic range to fall within the standard curve of the intended assay. ELISA assays (Human Quantikine^®^, R and D Systems™) were used to measure concentrations of MMP-9 and TIMP-1 according to manufacturer’s instructions (dilution range 40-800-fold). Multiplex chemiluminescent assays [V-plex Human Pro-inflammatory cytokine panel, Meso Scale Discovery™ (MSD)] were used to measure concentrations of ten cytokines: IFN-γ, IL-1β, IL-2, IL-4, IL-6, IL-8, IL-10, IL-12, IL-13, and TNF-α according to the manufacturer’s instructions (dilution range 4-100-fold). All samples were run in duplicate. The inter-plate coefficient of variance (CV) value provided by the manufacturer was 8% for MMP-9, 4% for TIMP-1 (R and D Systems™) and for the cytokine multiplex plates the CV is typically <10% (MSD™). An intra-plate CV cut off ≤15% was used as the limit of acceptable variation between duplicates for these analyses.

Light microscopy was performed on 19 samples from PWLWH for whom a paired dry HVS was available to grade polymorphonuclear leucocyte count by ordinal scale: 0, 1–5, 6– 10, 11–20, 21–30, and 31+ per high-powered field over an average of 3 fields.

### DNA Extraction and 16S rRNA Gene Sequencing (Metataxonomics)

For PWLWH bacterial DNA was extracted from the HVS using a combination of enzymatic digestion and mechanical disruption of cell membranes and the QIAamp^®^ DNA Mini kit (Qiagen™), as previously described (MacIntyre et al., 2015). The V1-V2 hypervariable regions of the 16S rRNA gene were amplified with a fusion primer set that includes four different 28F primers chosen to improve detection of *Bifidobacteriales* and a 388R primer(24). The 28F-YM forward primer (5′-GAGTTTGATCNTGGCTCAG-3′) was mixed in a ratio of 4:1:1:1 with 28F *Borrellia* (5’-GAGTTTGATCCTGGCTTAG-3’), 28F *Chloroflex* (5′-GAATTTGATCTTGGTTCAG-3’) and 28F *Bifido* (5’-GGGTTCGATTCTGGCTCAG-3’) (RTL Genomics Amplicon Diversity Assay List). The forward primers included an Illumina i5 adapter (5′-AATGATACGGCGACCACCGAGATCTACAC-3′), an 8-base-pair (bp) bar code and primer pad (forward, 5′-TATGGTAATT-3′). The 388R reverse primer (5′-TGCTGCCTCCCGTAGGAGT-3′) was constructed with an Illumina i7 adapter (5′-CAAGCAGAAGACGGCATACGAGAT-3′), an 8-bp bar code, a primer pad (reverse, 5′-AGTCAGTCAG- 3′). The pair end multiplex sequencing was performed on an Illumina MiSeq™ platform (Illumina^®^ Inc.) at Research and Testing Laboratory (Lubbock, TX, USA).

#### Sequence Analysis

The MiSeq SOP pipeline and software package Mothur were used to analyse RNA sequence data. Highly similar amplicons were clustered into operational taxonomic units (OTUs) using the kmer searching method and the Silva bacterial database (www.arb-silva.de/). All OTUs had a taxonomic cut-off of ≥97%. Classification was performed using the Ribosomal Database Project (RDP) reference sequence files and the Wang method (Wang et al., 2007). The RDP MultiClassifier script was used for determination of OTUs (phylum to genus) and species level taxonomies were determined using USEARCH (Edgar, 2010). To account for potential bias introduced by differences in sequence depth, samples were rarefied to the smallest OTU read count (n= 7738) and proportion of total reads calculated for each sample to generate species level abundance.

### Statistical Analyses

#### Cross-Sectional 2^nd^ and 3^rd^ Trimester Comparison by HIV Status

CVF from 50 PWLWH and 12 HUPW were available for cross-sectional analyses, each participant contributed one sample per trimester group. Maternal demographics were compared by Welch, Mann-Whitney U and Fisher Exact tests according to variable type, data distribution, variance, and group size. Mean MMP-9, TIMP-1, MMP-9/TIMP-1 ratio and cytokine concentrations were compared in a cross-section of second trimester (PWLWH n=47, HUPW n=10) and third trimester samples (PWLWH n=33, HUPW n= 9) by HIV status and cART exposure using Welch tests and ANOVA.

#### Longitudinal Analyses in PWLWH

Using longitudinal data from PWLWH alone (108 samples) associations of polymorphonuclear leucocyte count, cytokine concentrations and bacterial genera abundance with MMP-9, TIMP-1 concentrations and their ratio were explored initially by Spearman’s correlation and then partial correlation, adjusting for maternal age, race and BMI. To identify potential indirect effects between MMP-9, TIMP-1 and bacterial genera abundance, key cytokines were then included in partial correlation as covariates to test if the associations changed. A Bonferroni adjustment of p=0.01 was used as the threshold of significance to account for multiple comparison. To retain sensitivity p-values > 0.01 < 0.05 were considered as a trend.

Significant correlations were then characterised by hierarchical multiple linear regression adjusting for confounders, with patient ID included as a random effect and predictors inputted as second factors. Predicted models of the inter-relationship between MMP-9, TIMP-1 with key bacterial species and cytokines were postulated from regression results.

Mediation analyses were used to investigate whether the predicted relationship between *Gardnerella* and *Lactobacillus* genera and MMP-9 was mediated by indirect effects of key inflammatory cytokines or TIMP-1 [PROCESS macro, Model 4, version 4.0 (Preacher and Hayes, 2008)]. The 95% bootstrapped confidence interval for indirect effects is based on 1000 samples and considered significant if the bootstrapped confidence intervals did not cross zero. All analyses were performed in statistical software package, SPSS (version 27; IBM, Armonk NY, USA).

## Results

### Study Participants Characteristics

PWLWH and HUPW were of similar age (median 35 and 33 years respectively), see **Table 1**. The predominant race of PWLWH was Black (82%) whereas Caucasian race was the most common in HUPW (58%). PWLWH had higher median body-mass index (BMI) and lower CD4 cell counts than HUPW. There was no difference in gestational age at delivery and proportion of women delivering prematurely between PWLWH and HUPW in this sub-analysis (5 (10%) and 1 (8%), p=0.68), see **Table 1**.

**Table 1:** Maternal Demographics by HIV status.

Characteristic	PWLWH n=50	HUPW =12	P value
Age/years			
[Median (range)]	35 (21-45)	33 (20-43)	0.205
Ethnicity [n (%)]			<0.001
			
Caucasian	4 (10)	7 (58)	
Black	41 (82)	3 (25)	
Other	5 (8)	2 (18)	
BMI [Median (IQR)]	25 (22-30)	23 (20-25)	0.032
Baseline CD4 +/cells/mm^3^ [Median (IQR)]	620 (433-724)	970 (900-1390)	<0.001
Gestational age at delivery/weeks [Median (IQR)]	39 (38-40)	40 (38-41)	0.512
Birth outcome [n (%)]			
Term	42	27	0.682
Preterm	5	1	
Missing	3	1	
Birth weight/grams [Median (IQR)]	3190 (2908-3375)	3100 (2800-3200)	0.456

### cART Exposure in PWLWH

Thirty-six (72%) PWLWH conceived on cART and 14 initiated cART post conception (27%). cART comprised a backbone of two nucleoside analogue reverse transcriptase inhibitors and one of the following third agents: a Protease Inhibitor (PI) n = 17 (34%); a Non-Nucleoside Reverse Transcriptase Inhibitor (NNRTI) n = 20 (40%); an Integrase Strand Transfer Inhibitor (INSTI) n = 10 (20%) and a third NRTI n = 3 (6%).

### Cross-Sectional Analyses of CVF MMP-9, TIMP-1, and Cytokines in the Second and Third Trimester by HIV Status

#### CVF MMP-9, TIMP-1 and Pro-Inflammatory Cytokine Concentrations Are Higher in PWLWH Than HUPW

PWLWH had a higher mean concentration of MMP-9 in the second and third trimesters, although this did not reach significance in the third trimester (4 fold higher, p<0.001 and 1.5 fold, p=0.213 respectively), see **Table 2**. PWLWH had a higher mean concentration of TIMP-1 in the second and third trimesters (12 fold higher, p=0.035 and 3 fold higher, p=0.044 respectively). There was no statistical difference observed in MMP-9/TIMP-1 ratio by HIV status in the second and third trimester but a non-statistical trend towards an increase in ratio between second and third trimesters for PWLWH (1.7 fold higher, p=0.276) but not HUPW was observed, see **Table 2**.

**Table 2:** Mean MMP-9, TIMP-1 and MMP-9/TIMP-1 ratio in second and third trimesters by HIV status.

Group	2^nd^ trim MMP-9 ng/mL	3^rd^ trim MMP-9 ng/mL	2^nd^ trim TIMP-1 ng/mL	3^rd^ trim TIMP-1 ng/mL	2^nd^ trim MMP-9/TIMP-1 ratio	3^rd^ trim MMP-9/TIMP-1 ratio
PWLWH	2823 (1740-3906)	3289 (2074-4490)	230 (36-425)	135 (68-172)	114 (45-183)	437 (-172-1046
HUPW	767 (308-1225)	2241 (980-3502)	20 (2-37)	54 (6-102)	201 (-117-519)	190 (-49-429)
P value	<0.001	0.207	0.035	0.044	0.561	0.439

Second trimester analyses: PWLWH n=47, HUPW=10; Third trimester analyses: PWLWH n=33, HUPW= 9. Protein concentrations are given as geometric mean (95%CI).

PWLWH had higher concentrations of most measured CVF cytokines in the cross-section of second and third trimester samples, see **Supplementary Figures 1A, B**. The CVF cytokines with the most marked difference by HIV status in both trimesters were: IL-1β (19-22 fold higher, 2^nd^ p=0.002, 3^rd^ p=0.003); IL-8 (13-16-fold higher, 2^nd^ p<0.001, 3^rd^ p<0.001); IL-12 (8-9 fold higher, 2^nd^ p<0.001, 3^rd^ p<0.001) and TNF-α (15-27 fold higher, 2^nd^ p<0.001, 3^rd^ p=0.002), see **Table 3**.

**Table 3:** Mean IL-1β, IL-8, IL-12 and TNF-α in second and third trimesters by HIV status.

Group	2^nd^ trim IL-1β pg/mL	3^rd^ trim IL-1β pg/mL	2^nd^ trim IL-8 pg/mL	3^rd^ trim IL-8 pg/mL	2^nd^ trim IL-12 pg/mL	3^rd^ trim IL-12 pg/mL	2^nd^ trim TNF-α pg/mL	3^rd^ trim TNF-α pg/mL
PWLWH	10161 (4202-16118)	15930 (6519-25341)	95513 (65149-125877)	89231 (60837-117625)	63 (43-83)	124 (82-165)	291 (175-406)	493 (205-781)
HUPW	458 (81-836)	846 (62-1630)	6065 (-82-12213)	6819 (523-13115)	7 (-7-21)	15 (-16-47)	19 (-19-57)	18 (-15-51)
P value	0.002	0.003	<0.001	<0.001	<0.001	<0.001	<0.001	0.002

Second trimester analyses: PWLWH n=47, HUPW=10; Third trimester analyses: PWLWH n=33, HUPW= 9. Protein concentrations are given as geometric mean (95%CI).

#### CVF MMP-9, TIMP-1 and Pro-Inflammatory Cytokine Concentrations in PWLWH Do Not Differ by cART Exposure or Pre-Term Birth

In PWLWH no statistical differences in CVF MMP-9, TIMP-1 concentration or MMP-9/TIMP-1 ratio were observed by cART timing or class of the third drug, see **Supplementary Figure 2**. CVF cytokines: IL-1β, IL-8, IL-12 and TNF-α concentrations in PWLWH did not differ by cART timing or class of third ART drug, see **Supplementary Figures 3A, B**. There was no difference in MMP-9, TIMP-1 or CVF cytokines: IL-1β, IL-8, IL-12 and TNF-α by prematurity in either second or third trimesters, see **Supplementary Tables 1, 2**.

### Longitudinal Analyses in PWLWH

#### CVF MMP-9 Positively Correlates With TIMP-1, Polymorphonuclear Leucocytes, Pro-Inflammatory Cytokines and Vaginal Pathobiont Abundance in PWLWH

**Figure 1:**
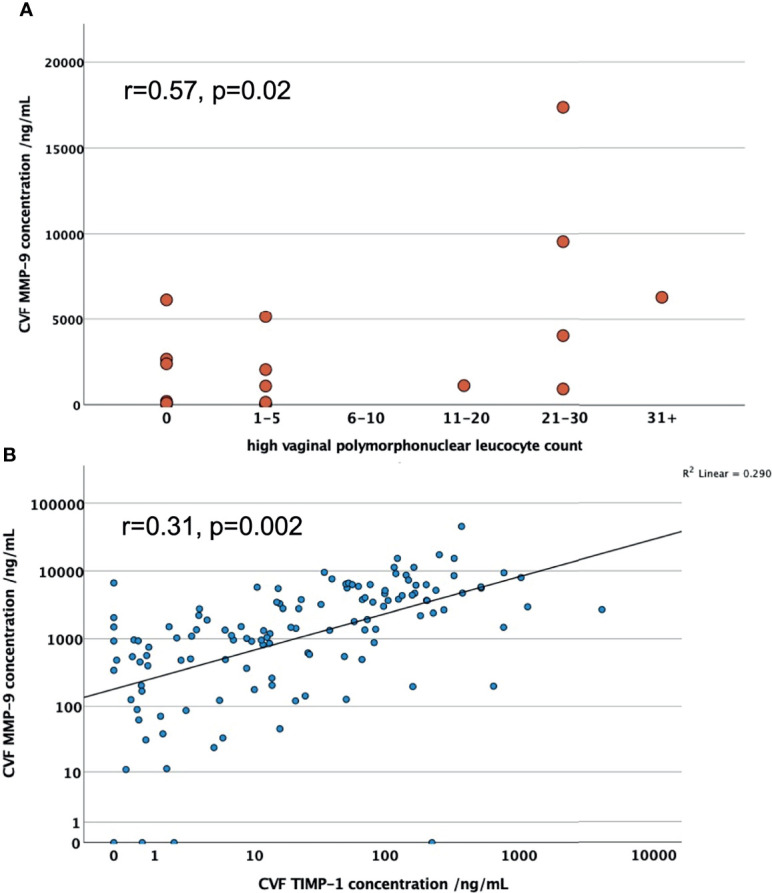
**(A)** Scatter graphs to explore correlation of CVF MMP-9 concentrations with high vaginal polymorphonuclear leucocyte counts in PWLWH; **(B)** Scatter graph to explore correlation between CVF MMP-9 and TIMP-1 concentrations in PWLWH. r = correlation co-efficient, controlled for maternal age, BMI and ethnicity.

In PWLWH CVF MMP-9 positively correlated with polymorphonuclear leucocyte count (r=0.57, n=19, p=0.02), see **Figure 1A**, and TIMP-1 concentration (r=0.31, n=108, p=0.002), after adjusting for maternal age, BMI and ethnicity, see **Figure 1B**. There was no statistical correlation of MMP-9 with gestational age at sampling (r=0.008, n=108, p=0.935).

**Figure 2:**
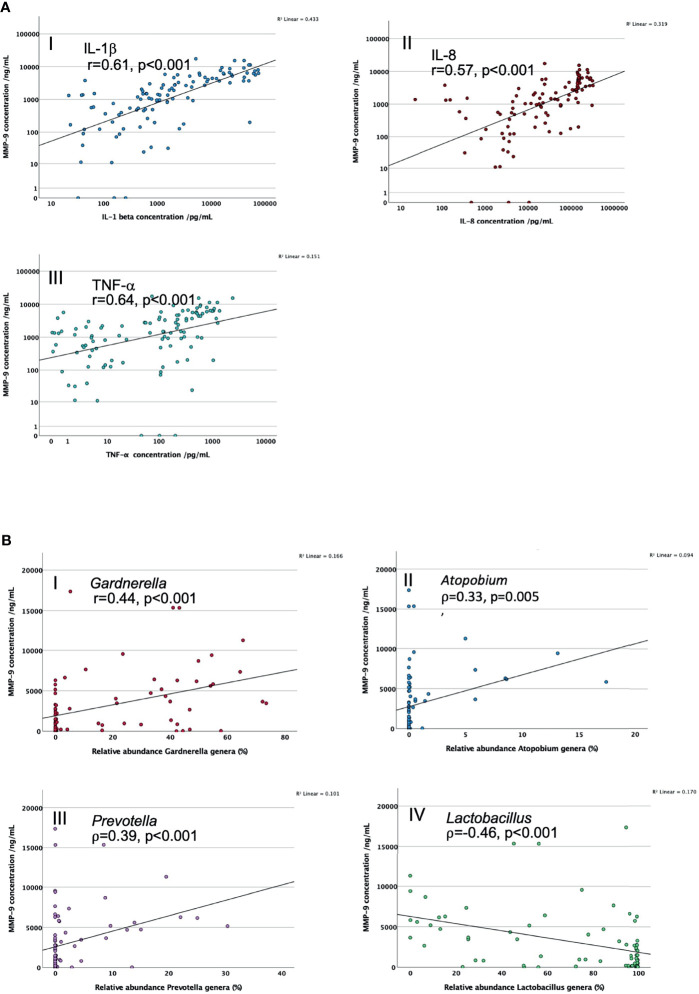
**(A)** Scatter graphs to explore correlation of CVF MMP-9 concentrations with key CVF cytokine concentrations. I. IL-1β; II. IL-8; III. TNF-α. **(B)** Scatter graphs to explore correlation of CVF MMP-9 concentrations with proportions of key vaginal bacterial genera. I. *Gardnerella;* II*. Atopobium;* III. *Prevotella;* IV. *Lactobacillus.* r = correlation co-efficient, controlled for maternal age, BMI and ethnicity.

MMP-9 positively correlated with IL-1β (r=0.61, n=91, p<0.001), IL-8 (r=0.57, n-91, p<0.001) and TNF-α (r=0.64, n=91, p<0.001), after adjusting for maternal age, BMI and ethnicity, see **Figure 2A**. Mean abundance of adverse anaerobic pathobionts also correlated positively with MMP-9 (p ≤ 0.005): *Gardnerella* (r=0.44, n=77, p<0.001), *Atopobium* (r=0.33, n=77, p=0.005), and *Prevotella* genera (r=0.39, n=77, p<0.001), see **Figure 2B**. Conversely mean proportion of *Lactobacillus* genera negatively correlated with MMP-9 (r= -0.46, n=77, p<0.001).

When partial correlation was controlled for cytokine IL-1β the associations between MMP-9 and key bacterial genera abundance were no longer significant (*Gardnerella:* r=0.10, p=0.433; *Atopobium*: r=0.15, p=0.225; *Prevotella*: r=-0.11, p=0.358 and *Lactobacillus*: r=-0.13, p=0.285). When partial correlation was controlled for IL-8 there was a lowering of the correlation efficient with *Gardnerella* (r=0.37, p=0.002) and *Lactobacillus* genera (r=-0.36, p<0.001) but the associations retained significance, the association with *Atopobium* (r=0.29, p=0.016) or *Prevotella* (r=0.23, p=0.056) were lost. When partial correlation was controlled for TNF-α there was a reduction of the correlation efficient with *Lactobacillus* (r=-0.26, p=0.034), *Gardnerella* (r=0.20, p=0.106)*, Atopobium* (r=0.21, p=0.088) and *Prevotella* genera (r=0.23, p=0.057).

#### CVF TIMP-1 Positively Correlates With Pro-Inflammatory Cytokines and Vaginal Pathobiont Abundance in PWLWH

**Figure 3:**
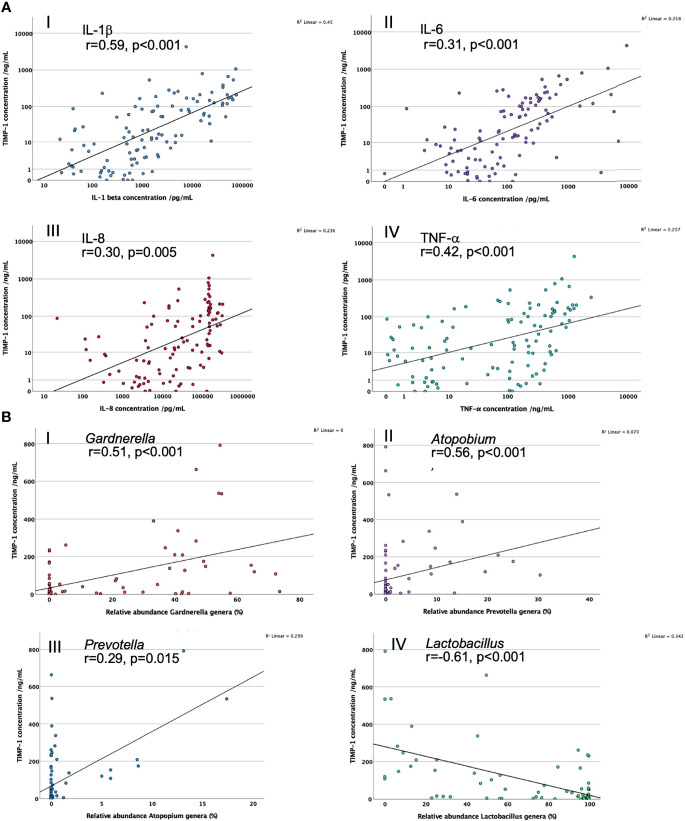
**(A)** Scatter graphs to explore correlation of CVF MMP-9 concentrations with key CVF cytokine concentrations. I. IL-1β; II. IL-6; III. IL-8; IV.TNF-α. **(B)** Scatter graphs to explore correlation of CVF MMP-9 concentrations with proportions of key vaginal bacterial genera. I. *Gardnerella;* II*. Atopobium;* III. *Prevotella;* IV. *Lactobacillus.* r = correlation co-efficient, controlled for maternal age, BMI and ethnicity.

In PWLWH TIMP-1 positively correlated with IL-1β (r=0.59, n=91, p<0.001), IL-6 (r=0.31, n=91, p=0.004), IL-8 (r=0.30, n=91, p=0.005) and TNF-α (r=0.42, n=91, p<0.001) after controlling for maternal age, BMI and ethnicity, see **Figure 3A**. Mean abundance of adverse anaerobic pathobionts correlated positively with TIMP-1 (p<0.02): *Gardnerella* (r=0.51, n=77, p<0.001), *Atopobium* (r=0.56, n=77, p<0.001) and *Prevotella* (r=0.29, n=77, p=0.015), after controlling for maternal age, BMI and ethnicity, see **Figure 3B**. Conversely, mean proportion of *Lactobacillus* genera negatively correlated with TIMP-1 (r=-0.61, n=77, p<0.001). There was no statistical correlation of TIMP-1 with polymorphonuclear leucocytes (r=-0.23, n=19, p=0.400) or gestational age at sampling (r=-0.15, n=108, p=0.146).

When partial correlation between TIMP-1 and key bacterial genera abundance was controlled for cytokine IL-1β, there was a substantial reduction in associations (*Gardnerella:* r=0.23, p=0.058; *Atopobium*: r=0.46, p<0.001; *Prevotella*: r=-0.03, p=0.829 and *Lactobacillus*: r=-0.39, p=0.001). When partial correlation was controlled for IL-8 there was minimal change of the correlation efficient with *Gardnerella* (r=0.47, p<0.001), *Atopobium* (r=0.54, p<0.001) and *Lactobacillus* genera (r=-0.57, p<0.001) but the association with *Prevotella* was lost (r=0.20, p=0.109). When partial correlation was controlled for TNF-α there was a moderate reduction of the correlation efficient with *Gardnerella* (r=0.39, p=0.001)*, Atopobium* (r=0.51, p<0.001), *Prevotella* genera (r=0.16, p=0.187) and *Lactobacillus* (r=-0.52, p<0.001).

When TIMP-1 was included in partial correlation between MMP-9 and bacterial abundance, the strength of the correlation co-efficients were lowered, some of which retained significance (*Gardnerella:* r=0.35, p=0.003; *Atopobium*: r=0.20, p=0.096; *Prevotella*: r=0.34, p=004 and *Lactobacillus*: r=-0.36, p=0.002).

#### CVF MMP-9/TIMP-1 Ratio Positively Correlates With Abundance of Vaginal *Lactobacillus* Genera and Negatively Correlates With *Gardnerella* Genera and Pro-Inflammatory Cytokines in PWLWH

**Figure 4:**
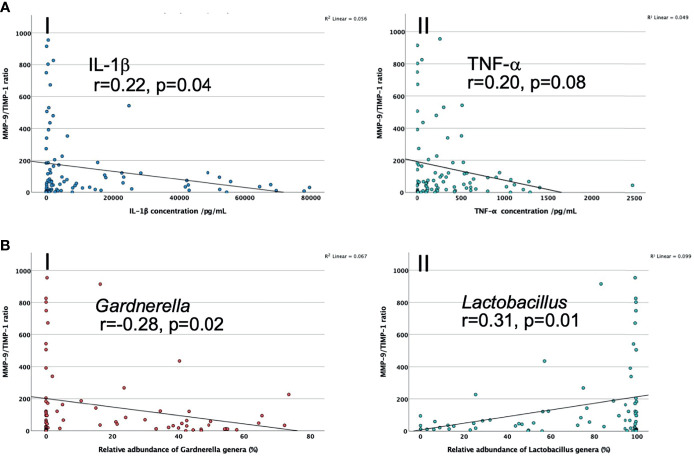
**(A)** Scatter graphs to explore correlation of CVF MMP-9/TIMP-1 ratio with I. IL-1β; II.TNF-α in PWLWH; **(B)** Scatter graph to show the association between CVF MMP-9/TIMP-1 ratio and I. *Gardnerella* genera*;* II. *Lactobacillus* genera in PWLWH. r = correlation co-efficient, controlled for maternal age, BMI and ethnicity.

There was a negative association between MMP-9/TIMP-1 ratio and IL-1β (r=-0.22, n=91, p=0.044) and TNF-α (r=-0.20, n=91, p=0.076), after controlling for maternal age, BMI and ethnicity in PWLWH, see **Figure 4A**. There was also a trend towards a negative correlation between MMP-9/TIMP-1 ratio with relative abundance of *Gardnerella* genera (r=-0.28, p=0.02), and a positive association with *Lactobacillus* genera (r=0.31, p=0.01), see **Figure 4B**.

**Figure 5:**
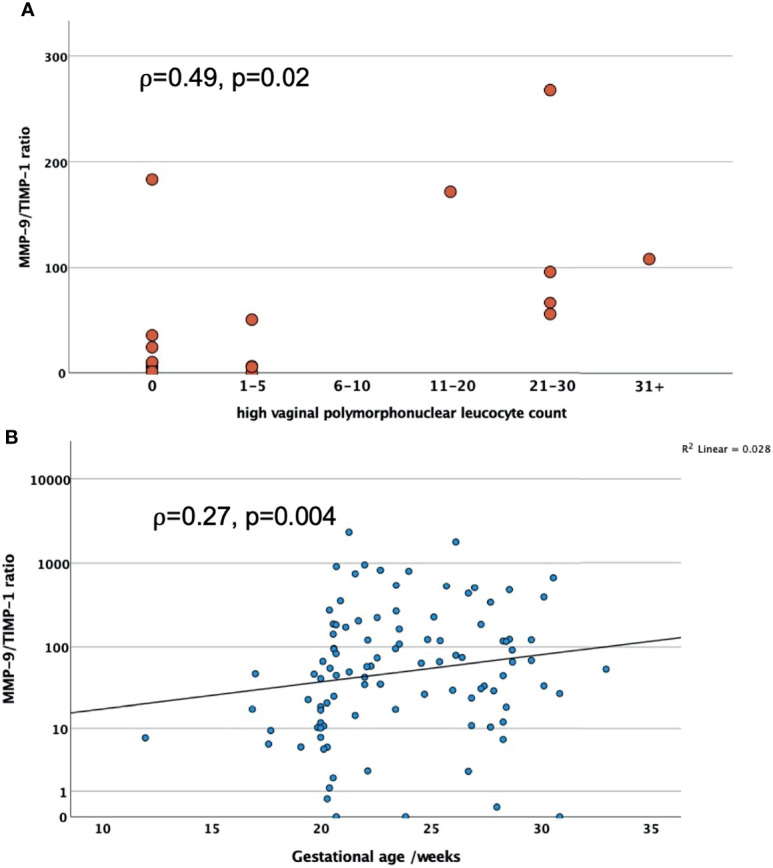
**(A)** Scatter graphs to explore correlation of CVF MMP-9/TIMP-1 ratio with polymorphonuclear leucocyte counts in PWLWH; **(B)** Scatter graph to show the association between CVF MMP-9/TIMP-1 ratio and gestational age at sampling in PWLWH ⍴ = rho.

Spearman’s correlation showed MMP-9/TIMP-1 ratio was positively associated with polymorphonuclear leucocyte count (rho=0.49, n=19 p=0.02) and gestational age at sampling (rho=0.27, n=108, p=0.004), see **Figure 5A, B**. The significance of these correlations were lost after controlling for maternal age, BMI and ethnicity: polymorphonuclear leucocyte count (r=0.36, n=19, p=0.184) and gestational age at sampling (r=0.005, n=108, p=0.603).

### Modelling of the Relationship Between Vaginal Bacteria, Cytokines, TIMP-1 and MMP-9 in PWLWH

To explore the relationship between MMP-9, TIMP-1, bacterial genera and IL-1β, hierarchical multiple linear regression was performed. The first step of the model was adjusted for potential confounders: maternal age, ethnicity and BMI, with patient ID included as a random effect and MMP-9, TIMP-1, IL-1β, *Gardnerella* and *Lactobacillus* genera abundance inputted as a second factors. In step one maternal age, ethnicity and BMI explained 5% of the variance in CVF MMP-9 concentration. After entry of predictor variables the total variance of the model as a whole was 47%, F (8, 64) =7.02, p<0.001. In the final model, only two measures were statistically significant with IL-1β recording a higher beta value (b=0.68, p<0.001) than TIMP-1(b=-0.29, p=0.039).

**Figure 6:**
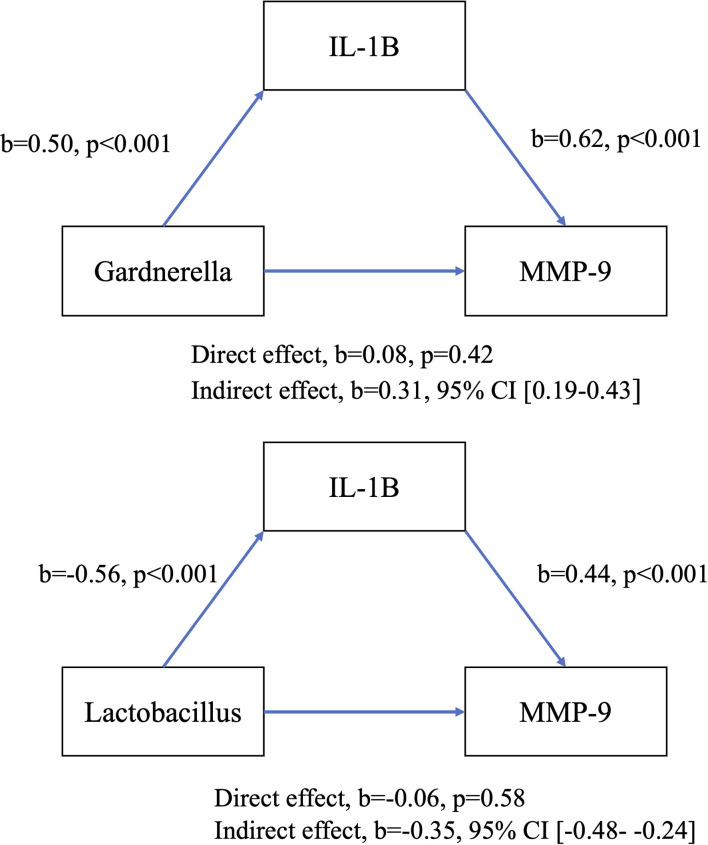
Model of direct and indirect relationships between MMP-9, key bacterial genera and IL-1β.

**Figure 7:**
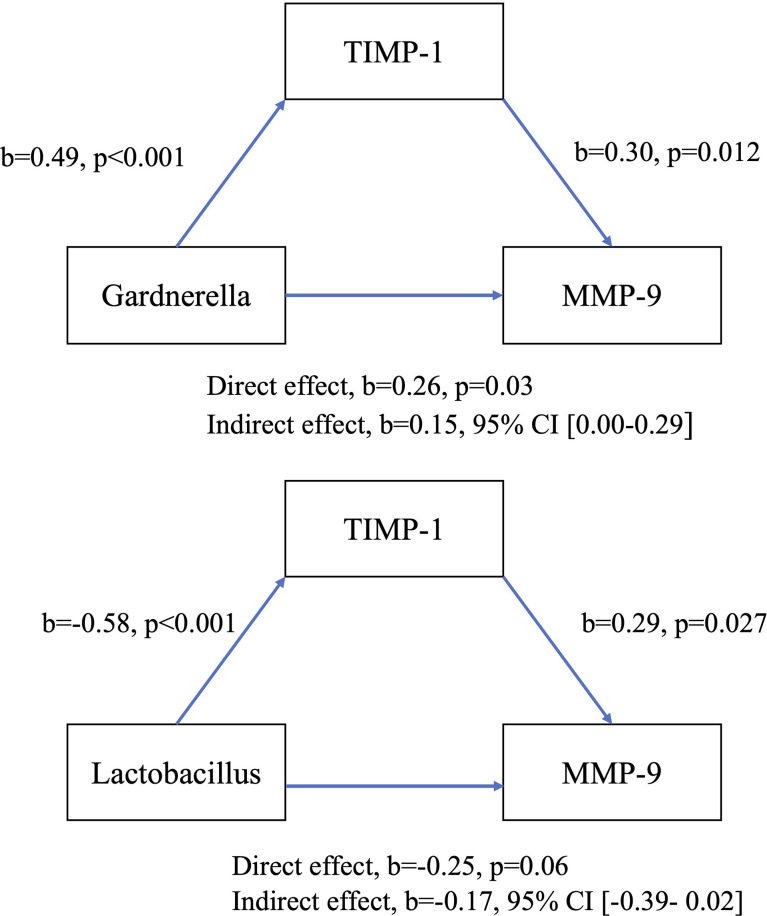
Model of direct and indirect relationship between MMP-9, key bacterial genera and TIMP-1.

Based on the significant associations identified in the preceding analyses, four models of mediation between vaginal bacterial genera and MMP-9 with indirect effect of IL-1β mediation and TIMP-1 mediation were proposed, see **Figure 6** and **7**. There was a significant indirect effect of vaginal *Gardnerella* abundance on CVF MMP-9 concentration through IL-1β consistent with mediation, b=0.31 BCa CI [0.19-0.43], see **Figure 6**. There was a significant indirect effect of vaginal *Lactobacillus* abundance on CVF MMP-9 concentration through IL-1β consistent with mediation, b=-0.35 BCa CI [-0.48- -0.23]. When exploring mediation of TIMP-1, there was no significant indirect effect on the relationship between either *Gardnerella* or *Lactobacillus* on MMP-9 concentrations, see **Figure 7**.

## Discussion

The main findings of these exploratory analyses include that PWLWH have high CVF concentrations of MMP-9, TIMP-1, pro-inflammatory cytokines and MMP-9/TIMP-1 ratios in comparison to the HUPW participants. In PWLWH, MMP-9 and TIMP-1 were highly correlated and MMP-9/TIMP-1 ratio increased with gestational age at sampling. CVF MMP-9 and TIMP-1 concentrations strongly correlated with vaginal proportions of anaerobic genera: *Gardnerella; Atopobium* and *Prevotella* and key inflammatory cytokines: IL-1β, IL-8 and TNF-α. Higher vaginal abundance of *Lactobacillus* genera associated with lower MMP-9 concentrations. Both MMP-9 and MMP-9/TIMP-1 ratio positively correlated with vaginal polymorphonuclear leucocyte counts. Exploration of indirect effects between MMP-9, *Gardnerella* and *Lactobacillus* genera and IL-1β indicate full mediation suggesting the association between vaginal bacterial and this interstitial collagenase are likely to be driven by changes in expression of this key pro-inflammatory cytokine. TIMP-1 was not shown to mediate the relationship between bacteria and MMP-9 in these data. Within this sub-analysis of PWLWH and HUPW who donated CVF to the HIV PTB study, there was no difference in expression of MMP-9 or TIMP-1 by prematurity, but numbers were small and unlikely to be powered to show such a difference.

The concentration of MMP-9 observed in the lower FGT of PWLWH in both second and third trimesters was much higher than previously published values of expression in serum of HUPW (Sorokin et al., 2010; Tency et al., 2012; Chen and Khalil, 2017), amniotic fluid of labouring and non-labouring HUPW [(Maymon et al., 2000; Locksmith et al., 2001, (Maymon et al., 2000; Locksmith et al., 2001; Myntti et al., 2017)] and the cervical mucous from HUPW (Becher et al., 2010). Becher and colleagues explored the expression of MMP-9, TIMP-1 and IL-8 in the cervical mucous plug (CMP) in pregnancy and found that MMP-9 and IL-8 expression was greatest at the distal portion of the CMP closest to the vagina (Becher et al., 2010). They also demonstrated that MMP-9, IL-8 but not TIMP-1 concentrations increased from early to late pregnancy and were higher in women labouring preterm compared to term. MMP-9 and TIMP-1 concentrations in our analyses of HUPW were 200 and 2-fold higher in the second trimester than those observed in CMP by Becher and colleagues in early pregnancy, with a positive CVF MMP-9/TIMP-1 ratio compared to an inverse ratio observed in Becher’s CMP samples. This is likely to be the result of an addition vaginal source of MMP-9 and differences in sample handling including the use of protease inhibitors. In addition, Becher and colleagues did not show a relationship between MMP-9 and vaginal bacteria but considered a limited number of pathobionts using less sensitive culture methods.

MMP-9 is known to be expressed by many cells at the maternal foetal interface including: neutrophils; macrophages; NK cells; vascular endothelial cells of the decidua; cervical fibroblasts and epithelial cells (Lockwood et al., 2008; Naruse et al., 2009; Gonzalez et al., 2011; Ulrich et al., 2019). Gonzalez and colleagues demonstrated that in PTB macrophages are the main source of MMP-9 at the cervix, whereas MMP-9 production at term is non-leucocyte dependant (Gonzalez et al., 2011). We hypothesise that neutrophils and macrophages are the predominant source of MMP-9 in the lower FGT of PWLWH and MMP-9 expression in the cervical environment is elevated as a result of an inflammatory response to vaginal microbiota composition, supported by the observed associations with polymorphonuclear leucocytes. Further in-depth modelling of the relationship with local innate immune cells in this cohort was limited by the small numbers for which matched vaginal polymorphonuclear leucocyte counts were available.

MMP-9 concentrations in CVF increase with cervical ripening at term (Choi et al., 2009) and are proposed to enable cervical effacement and dilatation through remodelling of the ECM (Peltier, 2003; Challis et al., 2009). In addition to its ECM effects, MMP-9 has a role processing other molecules including inflammatory cytokines with a demonstrated role in term and preterm labour: IL-8 and IL-1β. The correlations observed in these data between key inflammatory cytokines and MMP-9 may be the result of MMP-9 cleavage of these chemokines and cytokines or as a result of upregulation by downstream signalling of the innate immune response to vaginal pathobionts or both. The reciprocal upregulation observed in TIMP-1 may be the result of positive feedback and homeostatic regulation. TIMP-1 upregulation was not directly proportional to that seen with MMP-9 and hence there was an increase in MMP-9/TIMP-1 ratio across gestation, which could result in a balance that favours collagen IV degradation in ECM.

These analyses are the first to demonstrate the association between vaginal anaerobes, inflammation, and ECM remodelling proteases in PWLWH and adds to the body of evidence for an ascending infection model of PTB with a vaginal source and associated inflammatory response at the cervix, lowering the threshold for early initiation of labour. It must be borne in mind that associations do not imply causality and HIV-associated PTB is likely multi-factorial, including a role of cART which may modulate some of these pathways. HIV Protease Inhibitors, the most implicated class of antiretrovirals, are purported to not affect extracellular proteases but could potentially affect inhibitory protein activity. No demonstrable difference in expression of MMP-9 and TIMP-1 was observed by class of third agent in this small sample which may be the result of type 2 error. cART may also exert its effects directly on the vaginal microbiota (Ray et al., 2021). Genetic polymorphisms may also explain the elevated expression of MMP-9, 82% of whom were of Black race (Pereza et al., 2014; Pandey and Awasthi, 2020). The racial heterogeneity of this cohort may bias conclusions comparing by HIV status however analyses within PWLWH were adjusted for ethnicity and remain valid. The ethnicity of the PWLWH cohort reflect the women receiving antenatal care in the UK, many of whom are African and Caribbean migrants and may be generalisable to women in other settings, given the majority of PWLWH reside in Africa (Unaids.org, 2018). The findings of this study would ideally be replicated in a larger cohort, powered to look at differences by prematurity and include racially matched controls. The role of racial disparity in PTB risk is in itself an important question and is likely to be the result of many interplaying factors including: prejudice, genetics, socioeconomics, healthcare access and stress influencing downstream factors such as background co-morbidities, neuroendocrine, infection, microbiota, and immune mediators (Braveman et al., 2021).

Altered angiogenesis at implantation and placentation has been postulated as a mechanism underlying HIV associated PTB and low birth weight (Conroy et al., 2017). MMP-9 is a key regulator of this process in normal pregnancy and dysregulated expression has been implicated in both pre-eclampsia and IUGR (Juanjuan Merchant et al., 2004; Świerczewski et al., 2012; Chen and Khalil, 2017; Ardiani et al., 2019), the former of which was rarely seen in PWLWH in the pre cART era (Wimalasundera et al., 2002). More recently a case control study in Cameroon compared MMP-9 expression in plasma collected from the placental intervillous space by HIV status and found no difference in expression between groups suggesting any differences in MMP-9 in PWLWH are unlikely to be placental in origin (Esemu et al., 2019).

Within these sub-analyses we did not show any difference in gestational age at delivery or expression of CVF MMP-9 and TIMP-1 by prematurity. The small sample size reduces power to observe these differences in PTB rates, which were seen in the main HIV PTB study cohort (Short, 2019; Short et al., 2021). There was however a moderate association between MMP-9/TIMP-1 ratio with gestational age that warrants further exploration and validation in a larger group, ideally with matched controls and exploring other network proteins such as EMMPRIN and Lipocalin-2, the transcriptome and presence of local immune cells.

In conclusion, we have shown vaginal anaerobes and key pro-inflammatory cytokines positively correlate with ECM modifying protease MMP-9 and its inhibitory protein TIMP-1 in PWLWH, the ratio of which increase in pregnancy and may result in cervical remodelling as one mechanism underlying HIV-associated PTB.

## Data Availability Statement

The datasets presented in this study can be found in online repositories. The names of the repository/repositories and accession number(s) can be found below: https://www.ebi.ac.uk/ena, PRJEB41429; ena-STUDY-CUMICRO-18-11-2020-15:38: 11:823-609.

## Ethics Statement

The studies involving human participants were reviewed and approved by NHS Health Research Authority National Research Ethics Service (NRES) (Ref 13/LO/0107). The patients/participants provided their written informed consent to participate in this study.

## Author Contributions

C-ES, PB, GT, and DM conceived and designed the study. Patient recruitment and sample collection were undertaken by C-ES and RQ. Experiments and data collection were performed by C-ES, RQ, XW, VP and YL. Data processing, analyses, and interpretation were performed by C-ES, AS, PB, GT, and DM. All figures and tables were generated by C-ES. C-ES wrote the first draft of the manuscript and all authors contributed critical revisions to the paper, interpretation of the results and approved the final version.

## Funding

This study was funded by the Wellcome Trust Clinical PhD Programme (C-ES, grant no. WT/102757/Z/13/Z, the Medical Research Council (DM, grant no. MR/L009226/1), the March of Dimes European Preterm Birth Research Centre at Imperial College London and the National Institute of Health Research (NIHR) Imperial Biomedical Research Centre (BRC) (PB and DM, grant no. P45272).

## Author Disclaimer

The views expressed are those of the authors and not necessarily those of the NHS, the NIHR, or the Department of Health.

## Conflict of Interest

PB reports personal fees and shares and stock ownership in ObsEva Pharmaceuticals, personal fees from GlaxoSmithKline that are both outside the submitted work. PB and DM have a patent for microRNA markers to predict cervical shortening and preterm birth issued again outside of the submitted work.

The remaining authors declare that the research was conducted in the absence of any commercial or financial relationships that could be construed as a potential conflict of interest.

## Publisher’s Note

All claims expressed in this article are solely those of the authors and do not necessarily represent those of their affiliated organizations, or those of the publisher, the editors and the reviewers. Any product that may be evaluated in this article, or claim that may be made by its manufacturer, is not guaranteed or endorsed by the publisher.
