# Supplementary material for: Vaginal Microbiota, Genital Inflammation and Extracellular Matrix Remodelling Collagenase: MMP-9 in Pregnant Women With HIV, a Potential Preterm Birth Mechanism Warranting Further Exploration
Source: Front Cell Infect Microbiol. 2021 Nov 29;11:750103. doi: 10.3389/fcimb.2021.750103 (PMC8667959; doi:10.3389/fcimb.2021.750103)
Supplement: Supplementary file 1 [file DataSheet_1.docx]

Supplementary Material


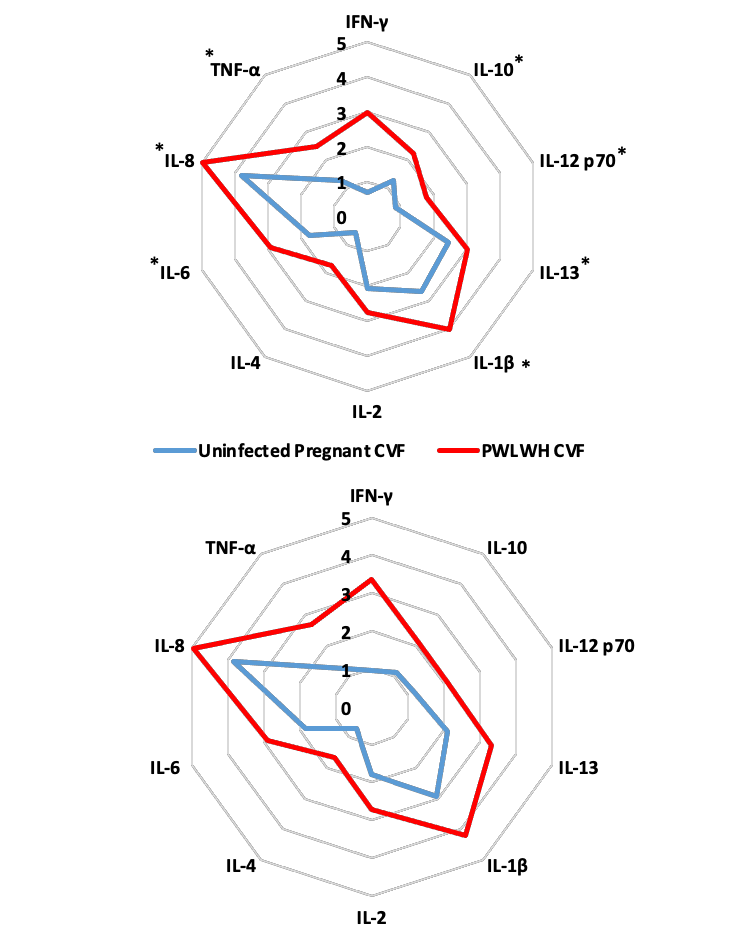


Supplementary figure 1. Spider-chart to demonstrate log^10^ mean CVF cytokine concentration by HIV status. A: Second trimester; B: Third trimester * p<0.01

Supplementary Figure 2A. Bar charts to demonstrate mean second trimester CVF MMP-9 concentration in PWLWH with 95% confidence interval by: I. cART timing in relation to conception and II. class of third cART agent. Uninfected pregnant women data presented for comparative reference.

2B. Bar charts to demonstrate mean second trimester CVF TIMP-1 concentration in PWLWH by: I. cART timing in relation to conception and II. class of third cART agent.

2C. Bar charts to demonstrate mean second trimester CVF MMP-9/TIMP-1 ratio in PWLWH by: I. cART timing in relation to conception and II. class of third cART agent

PI= Protease Inhibitor; NNRTI= Non Nucleoside Reverse Transcriptase Inhibitor; INSTI= Integrase Strand Transfer Inhibitor; NRTI= Nucleoside Reverse Transcriptase Inhibitor.

Supplementary Figure 3A. Bar charts to demonstrate mean second trimester CVF IL-1B concentration in PWLWH with 95% confidence interval by: I. cART timing in relation to conception and II. class of third cART agent. Uninfected pregnant women presented for comparative reference.

3B. Bar charts to demonstrate mean second trimester CVF IL-8 concentration in PWLWH by: I. cART timing in relation to conception and II. class of third cART agent.

3C. Bar charts to demonstrate mean second trimester CVF IL-12 in PWLWH by: I. cART timing in relation to conception and II. class of third cART agent

3D. Bar charts to demonstrate mean second trimester CVF TNF-α in PWLWH by: I. cART timing in relation to conception and II. class of third cART agent

PI= Protease Inhibitor; NNRTI= Non Nucleoside Reverse Transcriptase Inhibitor; INSTI= Integrase Strand Transfer Inhibitor; NRTI= Nucleoside Reverse Transcriptase Inhibitor.

Supplementary Table 1. Mean MMP-9, TIMP-1 and MMP-9/TIMP-1 ratio in second and third trimesters by Prematurity.

| **Group** | **2^nd^ trim**  **MMP-9 /ng/mL** | **3^rd^ trim**  **MMP-9**  **/ng/mL** | **2^nd^ trim**  **TIMP-1**  **/ng/mL** | **3^rd^ trim**  **TIMP-1**  **/ng/mL** | **2^nd^ trim**  **MMP-9/TIMP-1 ratio** | **3^rd^ trim**  **MMP-9/TIMP-1 ratio** |
| --- | --- | --- | --- | --- | --- | --- |
| Term | 2953 (1724-4183) | 3351. (1919-4785) | 242 (15-469) | 149 (69-230) | 119 (37-201) | 520 (-235 -1276) |
| PTB | 1357 (-339 -3054) | 883 (-153 -1919) | 211 (-202 -625) | 18 (-29 -65) | 89 (-57 -236) | 96 (-101 -293) |
| P value | 0.366 | 0.255 | 0.924 | 0.289 | 0.791 | 0.702 |

Second trimester analyses in PWLWH: Term =40, PTB =5; third trimester analyses: Term =28, PTB=3. Protein concentrations are given as geometric mean (95% CI)

Supplementary Table 2. Mean IL-1β, IL-8, IL-12 and TNF-α in second and third trimesters by Prematurity.

| **Group** | **2^nd^ trim**  **IL-1β**  **pg/mL** | **3^rd^ trim**  **IL-1β**  **pg/mL** | **2^nd^ trim**  **IL-8**  **pg/mL** | **3^rd^ trim**  **IL-8**  **pg/mL** | **2^nd^ trim**  **IL-12**  **pg/mL** | **3^rd^ trim**  **IL-12**  **pg/mL** | **2^nd^ trim**  **TNF-α**  **pg/mL** | **3^rd^ trim**  **TNF-α**  **pg/mL** |
| --- | --- | --- | --- | --- | --- | --- | --- | --- |
| Term  n=36 | 10962 (4069 -17856) | 16117 (5024 -27210) | 98038 (65953 -130122) | 86298 (54591 -118005) | 64  (43 -85) | 126  (78 -174) | 314  (184 -444) | 489  (136 -842) |
| PTB  n=4 | 4097  (-7378 -15573) | 1115  (-5069 -7300) | 77728  (-146399 -301855) | 15152  (-66787 -97092) | 56  (-103 - 215) | 139  (-1569-847) | 91  (-169 -352) | 212  (-2359 -2782) |
| P value | 0.512 | 0.415 | 0.700 | 0.182 | 0.820 | 0.877 | 0.264 | 0.635 |

Second trimester analyses in PWLWH: Term =36, PTB =4; third trimester analyses: Term =22, PTB=2. Protein concentrations are given as geometric mean (95% CI)
